# Retrospective observational study of HER2 immunohistochemistry in borderline breast cancer patients undergoing neoadjuvant therapy, with an emphasis on Group 2 (*HER2*/*CEP17* ratio ≥2.0, *HER2* copy number <4.0 signals/cell) cases

**DOI:** 10.1038/s41416-021-01351-8

**Published:** 2021-03-24

**Authors:** Emad A. Rakha, Islam M. Miligy, Cecily M. Quinn, Elena Provenzano, Abeer M. Shaaban, Caterina Marchiò, Michael S. Toss, Grace Gallagy, Ciara Murray, Janice Walshe, Ayaka Katayama, Karim Eldib, Nahla Badr, Bruce Tanchel, Rebecca Millican-Slater, Colin Purdie, Dave Purnell, Sarah E. Pinder, Ian O. Ellis, Andrew H. S. Lee

**Affiliations:** 1 Department of Histopathology, Nottingham University Hospitals, Nottingham, UK; 2 Nottingham Breast Cancer Research Centre, Division of Cancer and Stem Cells, School of Medicine, Nottingham City Hospital, The University of Nottingham, Nottingham, UK; 3 Histopathology Department, Faculty of Medicine, Menoufia University, Shebin El-Kom, Egypt; 4 Histopathology, Breast Check, Irish National Breast Screening Programme and St. Vincent’s University Hospital, Dublin, Ireland; 5 Department of Pathology, University of Cambridge, Cambridge, UK; 6 Queen Elizabeth Hospital Birmingham and Institute of Cancer and Genomic Sciences, The University of Birmingham, Birmingham, UK; 7 Department of Medical Sciences, University of Turin, Turin, Italy; 8 Pathology Unit, Candiolo Cancer Institute, FPO-IRCCS, Candiolo, Italy; 9 Discipline of Pathology, School of Medicine, Lambe Institute for Translational Research, National University of Ireland, Galway, Ireland; 10 Department of Histopathology, St James’s University Hospital, Leeds, UK; 11 Department of Breast Pathology, Ninewells Hospital and Medical School, Dundee, UK; 12 Histopathology Department, University Hospitals of Leicester, Leicester, UK; 13 Division of Cancer Studies, King’s College London, Guy’s Hospital, London, UK

**Keywords:** Breast cancer, Breast cancer

## Abstract

**Background:**

The ASCO/CAP guidance on HER2 testing in breast cancer (BC) has recently changed. Group 2 tumours with immunohistochemistry score 2+ and *HER2*/*CEP17* ratio ≥2.0 and *HER2* copy number <4.0 signals/cell were re-classified as HER2 negative. This study aims to examine the response of Group 2 tumours to neoadjuvant chemotherapy (NACT).

**Methods:**

749 BC cases were identified from 11 institutions. The association between HER2 groups and pathological complete response (pCR) was assessed.

**Results:**

54% of immunohistochemistry HER2 positive (score 3+) BCs showed pCR, compared to 19% of immunohistochemistry 2+ FISH amplified cases. 27% of Group 2 treated with HER2 targeted therapy achieved pCR, compared to 19 and 11% in the combined Groups 1 + 3 and Groups 4 + 5, respectively. No difference in pCR rates was identified between Group 2 and Group 1 or combined Groups 1 + 3. However, Group 2 response rate was higher than Groups 4 + 5 (*p* = 0.017).

**Conclusion:**

No difference in pCR was detected in tumours with a *HER2/*CEP17 ratio ≥2.0 and a HER2 score 2+ by IHC when stratified by *HER2* gene copy number. Our data suggest that ASCO/CAP HER2 Group 2 carcinomas should be evaluated further with respect to eligibility for HER2 targeted therapy.

## Background

Approximately 15–20% of newly diagnosed invasive breast cancers (BC) show human epidermal growth factor receptor 2 (HER2) protein overexpression, usually due to *HER2* gene amplification.^1–4^ HER2 overexpression is associated with a worse prognosis in patients who do not receive adjuvant systemic therapy and is predictive of response to several systemic therapies,^5^ in particular to HER2 targeted treatments.^6–9^ Hence, eligibility criteria based on HER2 status have been developed to optimise patient selection for these expensive and potentially toxic targeted agents and have evolved over time.^3,4,10^

The early trials of trastuzumab in metastatic BC enrolled patients with HER2 status defined using immunohistochemistry (IHC) assays alone and considered both 3+ and 2+ IHC scores as eligible for these trials.^4,6,7,11^ Subsequent analyses identified that only patients with HER2 positive BC, defined as IHC 3+ reactivity and/or *HER2* gene amplification confirmed by fluorescent in situ hybridisation (FISH), benefit from HER2 targeted therapies.^6,7,12–14^ The Food and Drug Administration (FDA) definition of HER2 positivity was updated to IHC 3+ or 2+ with *HER2* gene amplification (defined as *HER2*/chromosome enumeration probe 17 [*CEP17*] ratio ≥2.0, regardless of the *HER2* copy number).^13,15,16^ This definition was endorsed by the early HER2 guidelines in the United Kingdom (UK).^17,18^ Subsequent guidelines by the American Society of Clinical Oncology (ASCO)/College of American Pathologists (CAP) in 2007^10^ and 2013^4^ and the further UK 2015 update^19^ expanded the definition of positivity to include tumours with an average *HER2* gene copy number ≥6 signals/nucleus regardless of the *HER2*/*CEP17* ratio.

In 2018, ASCO/CAP published an update to refine the definition of cases showing mismatch between the *HER2/CEP17* ratio and *HER2* gene copy number, comprising ~4–15% of cases.^3^ Possible combinations of the ratio and gene copy number were classified into 5 groups (Table 1). Unlike Group 1 and Group 3, Group 2, defined as a *HER2/CEP17* ratio ≥2.0 with an average *HER2* copy number <4.0 signals per cell with IHC score of 2+, was re-classified from HER2 positive^4,19^ to HER2 negative.^3^ This change was based on the lack of substantive evidence for the efficacy of HER2-targeted therapy in such tumours in terms of survival benefit.^3,20^ These tumours are rare. In a study of 4331 tumours with known *HER2/CEP17* ratio, *HER2* copy number and HER2 IHC status, Press et al.^20^ identified only 35 Group 2 tumours, of which only three displayed an IHC 2+ score. Similar findings were observed by Ballard et al., who found 1.4% of tumours fell in this group they called ‘monosomy non-classical’ *HER2* amplified BC cases (ratio ≥ 2.0 with an average *HER2* copy number <4.0 signals per cell).^21^ The other two groups in their ‘non classical amplification category’ were designated as the ‘co-amplified group’ (ratio < 2.0, and average HER2 copy number/cell ≥6.0) and a ‘low amplified group’ (ratio ≥ 2.0 and average HER2 copy number/cell 4.0–5.9) and represented 0.8% and 2.1% of cases, respectively. The limited number of patients with such tumours in the first generation of adjuvant trastuzumab trials has been an obstacle to drawing definitive conclusions regarding their response to HER2 targeted therapy, and clinical data for this unusual category of cases are desperately required.

**Table 1:** Summary of the cohort including the distribution of the different HER2 groups and other variables.

Variables	HER2 IHC defined Groups							Total No. (%)
	IHC 2+ Group					IHC 3+ Group (positive control)	IHC 0/1+ Group (negative control)	
	Group 1 No. (%)	Group 2 No. (%)	Group 3 No. (%)	Group 4 No. (%)	Group 5 No. (%)	No. (%)	No. (%)	
*Total number*	121 (16)	46 (6)	13 (2)	84 (11)	106 (14)	146 (20)	233 (31)	749 (100)
*Tumour grade*								
1	1 (1)	1 (2)	1 (8)	2 (2)	3 (3)	2 (2)	1 (1)	11 (2)
2	63 (54)	25 (56)	5 (38)	39 (47)	69 (66)	83 (61)	130 (56)	414 (56)
3	53 (45)	19 (42)	7 (54)	42 (51)	32 (31)	52 (38)	100 (43)	305 (42)
*Oestrogen receptor*								
Negative	26 (21)	12 (26)	0 (0)	16 (19)	26 (25)	64 (44)	113 (49)	257 (35)
Positive	95 (79)	34 (74)	13 (100)	68 (81)	80 (75)	81 (56)	118 (51)	489 (65)
*HER2 targeted therapy given*								
No	13 (11)	5 (11)	5 (38)	84 (100)	106 (100)	8 (6)	233 (100)	454 (60)
Yes	108 (89)	41 (89)	8 (62)	0 (0)	0 (0)	130 (94)	0 (0)	295 (40)
*Response to neoadjuvant therapy*								
No response	5 (4)	2 (4)	0 (0)	5 (6)	17 (16)	10 (7)	37 (16)	76 (10)
Partial response	94 (78)	32 (70)	12 (92)	70 (83)	77 (73)	57 (39)	147 (63)	489 (65)
Pathological complete response	22 (18)	12 (26)	1 (8)	9 (11)	12 (11)	79 (54)	49 (21)	184 (25)
*HER2 gene copy number* (Mean)	7.40	3.40	6.92	4.63	2.56	12.57	–	–
Median (range)	7.6 (4.0–42.2)	3.4 (2.2–3.9)	6.5 (6.1–7.4)	4.6 (4.0–5.6)	2.6 (1.4-3.9)	10.8 (8.4–17.3)	–	–
*CEP17 copy number* (mean)	2.14	1.48	4.33	3.13	1.98	2.93	–	–
Median (range)	2.1 (1.2–5.7)	1.5 (1.0–1.9)	4.1 (3.2–5.5)	3.3 (2.2–4.9)	2.0 (1.2–12.3)	2.9 (2.6–3.5)	–	–

The cohort was classified into 5 categories (Group 1–5) for IHC 2+ cases according to the 2018 ASCO/CAP guideline as follows: Group 1 (IHC 2+, *HER/CEP* ratio ≥2.0; average *HER2* gene copy number ≥4.0), Group 2 (IHC 2+, *HER/CEP* ratio ≥2.0; average copy number <4.0), Group 3 (IHC 2+, *HER/CEP* ratio <2.0; average copy number ≥6.0), Group 4 (IHC 2+ *HER/CEP* ratio <2.0; average copy number 4.0–6.0), and Group 5 (IHC 2+, *HER/CEP* ratio <2.0; average *HER2* gene copy number <4.0). All tumours were selected from patients who received neoadjuvant therapy.

*IHC* immunohistochemistry.

In view of the low incidence of Group 2 tumours and the difficulty in identifying sufficient numbers to demonstrate a reliable survival benefit of targeted therapy, this study aimed to assess the behaviour of these tumours in the neoadjuvant setting as compared to the other well-defined groups, utilising a large multi-institutional cohort. Complete pathological response (pCR) to neoadjuvant therapy is a recognised surrogate for survival outcomes and, when compared to definite HER2 positive (3+) and HER2 negative (0/1+) groups, provides an indication of the underlying biology of these tumours with respect to the HER2 pathway activation and response to anti-HER2 therapy.

## Methods

### Study cohort

We analysed a retrospective cohort of 1374 BC patients who received neoadjuvant chemotherapy (NACT), with or without targeted therapy, and subsequent surgical resection in the years 2013–2019 from 11 institutions (Nottingham University Hospitals NHS Trust; Addenbrookes Hospital, Cambridge; University Hospitals Birmingham NHS Foundation Trust; University Hospitals of Leicester NHS Trust; St. Vincent’s University Hospital, Dublin; University Hospital Galway; St. Helens and Knowsley Teaching Hospital NHS Trust, Liverpool; Guy’s and St. Thomas’ Hospital, London; Ninewells Hospital, Dundee, Leeds Teaching Hospitals NHS Trust, University of Turin, Italy). Inclusion criteria included availability of data on *HER2* gene copy number and *HER2/CEP17* ratio, NACT and pathological response details with emphasis on Group 2 tumours. As control groups, some cases with IHC scores of HER2 3+ and 0/1+ were included (Table 1). Cases were identified and data collected from all centres based on these defined criteria to avoid sample bias.

Histological grade, details of oestrogen receptor (ER) status and treatment regimen received were also collected. pCR was defined as no residual invasive carcinoma in both the breast and axillary lymph nodes regardless of the presence of residual ductal carcinoma in situ (ypT0/is ypN0).^23^ All histopathological information was obtained from the original pathology reports. Anonymised data were analysed centrally. HER2 status was assessed using IHC and ISH, as described in the UK guidelines,^19^ with primary IHC, followed by ISH on all borderline (2+) cases. In cases with an unusual ISH pattern, such as the target study cohort, the existing UK guidelines mandate counting 60 instead of 20 cells. According to the IHC and ISH results, the tumours were classified into the ASCO/CAP HER2 Groups^3^ (Table 1). ER positivity were defined as nuclear staining of ≥1% of invasive tumour cells.^24^

Patients were considered eligible for HER2 targeted therapies if their tumours showed a HER2 IHC score of 3+, or 2+ with *HER2/CEP17* ratio ≥2.0 regardless of the *HER2* copy number (i.e. ASCO/CAP Groups 1 and 2) or if the *HER2* copy number was ≥6 (Group 3). For subgroup analysis in this study, patients were categorised according to HER2 status into (1) Groups 1, 2 and 3, and (2) Groups 4 and 5. Patients were subdivided into two groups based on the type of neoadjuvant therapy received: (1) chemotherapy in combination with HER2 targeted therapy, trastuzumab alone or with either pertuzumab or lapatinib and (2) patients who received chemotherapy only. Chemotherapy regimens given were in accordance with individual unit protocols and included anthracycline and taxane, anthracycline without taxane or non-anthracycline based regimens.

This study was approved by the Nottingham Research Tissue Bank Access Committee under the IRAS Project ID: 184265. Data were collected as fully anonymised.

### Statistical analysis

Statistical analysis was performed with SPSS (IBM SPSS Statistic, Version 24.0). Associations between clinicopathological variables and pCR were examined with Pearson Chi-square with Yates correction (*χ*^2^) or Fisher’s exact tests, as appropriate. Bonferroni correction for multiple testing was used to adjust *p* values. Proportional odds logistic regression test was used to adjust cofounders whenever needed. A two-tailed *p-*value < 0.05 was considered statistically significant.

## Results

Of the informative cases, 749 patients received NACT, with or without HER2 targeted therapy. This included 233 patients with negative HER2 status (IHC score 0 or 1+), and 516 patients with a HER2 IHC score of 2+ (*n* = 370) or 3+ (*n* = 146) (Table 1). There was a strong correlation between positive HER2 status (IHC 3+ and FISH-amplified Groups 1, 2 and 3) and response to NACT (*p* < 0.0001) and this correlation was maintained on stratifying based on ER status (*p* = 0.054 and *p* < 0.0001 in the ER negative and ER positive groups, respectively). BC patients with HER2 IHC score of 3+ achieved pCR in 54% of cases, compared to 19% of those with IHC score of 2+ with FISH amplification (*HER2/CEP17* ratio ≥2.0 or *HER2* gene copy number ≥6.0 (combined Groups 1, 2 + 3) (*p* < 0.0001) (Supplementary Table 1). The response to neoadjuvant therapy in group 2 was independent of other histological and treatment factors (Supplementary Table 2). This difference was significant in the subgroup of women with ER positive BC who received HER2 targeted therapy, here the IHC 3+ tumours showed a pCR rate of 58% compared to 15% in the IHC 2+ FISH amplified Groups (1, 2 and 3; 19/123) (*p* < 0.0001), but not in the ER negative subgroup (pCR rate of 53% for IHC 3+ versus 38% IHC 2+ amplified, (*p* = 0.68)) (Table 2). The magnitude of benefit from the addition of HER2 targeted therapy to chemotherapy was the same in both HER2 positive groups (IHC 3+ and the IHC 2+ FISH amplified) with a 2.3-fold increase in the pCR rate (24–56% and 9–21%, respectively) (Supplementary Table 1).

**Table 2:** Degree of pathological response in subgroups based on HER2 targeted therapy and oestrogen receptor status.

Variables	HER2 IHC defined groups							Total No. (%)	*p*-value	Adjusted *p* value (Bonferroni correction)
	IHC 2+ Group					IHC 3+ Group	IHC0/1+ Group			
	Group 1 No. (%)	Group 2 No. (%)	Group 3 No. (%)	Group 4 No. (%)	Group 5 No. (%)	Positive control No. (%)	Negative control No. (%)			
*Whole cohort*										
*HER2 Targeted therapy not offered*										
No response	2 (15)	0 (0)	0 (0)	5 (6)	17 (16)	1 (13)	37 (16)	62 (14)	**0.003**	**0.009**
Partial response	10 (77)	4 (80)	5 (100)	70 (83)	77 (73)	5 (63)	147 (63)	318 (70)		
Pathological complete response	1 (8)	1 (20)	0 (0)	9 (11)	12 (11)	2 (24)	49 (21)	74 (16)		
*HER2 Targeted therapy given*										
No response	3 (3)	2 (5)	0 (0)	0 (0)	0 (0)	9 (6)	0 (0)	14 (5)	**<0.0001**	**<0.0001**
Partial response	84 (78)	28 (68)	7 (88)	0 (0)	0 (0)	52 (38)	0 (0)	171 (58)		
Pathological complete response	21 (19)	11 (27)	1 (12)	0 (0)	0 (0)	77 (56)	0 (0)	110 (37)		
*Oestrogen receptor negative*										
No response	1 (4)	0 (0)	0 (0)	2 (12)	3 (11)	4 (6)	21 (19)	31 (12)	**0.030**	0.054
Partial response	15 (58)	7 (58)	0 (0)	11 (69)	16 (62)	26 (41)	58 (51)	131 (52)		
Pathological complete response	10 (38)	5 (42)	0 (0)	3 (19)	7 (27)	34 (53)	34 (30)	93 (36)		
*Oestrogen receptor positive*										
No response	4 (4)	2 (6)	0 (0)	3 (4)	14 (18)	6 (7)	16 (14)	45 (9)	**<0.0001**	**<0.0001**
Partial response	79 (83)	25 (73)	12 (92)	59 (87)	61 (76)	30 (37)	87 (74)	353 (72)		
Pathological complete response	12 (13)	7 (21)	1 (8)	6 (9)	5 (6)	45 (56)	15 (13)	91 (19)		
*HER2 targeted therapy treated cohort*										
*Oestrogen receptor negative*										
No response	1 (4)	0 (0)	0 (0)	–	–	3 (5)	–	4 (4)		
Partial response	13 (57)	7 (63)	0 (0)	–	–	25 (42)	–	45 (48)	0.281	0.680
Pathological complete response	9 (39)	4 (37)	0 (0)	–	–	32 (53)	–	45 (48)		
*Oestrogen receptor positive*										
No response	2 (2)	2 (7)	0 (0)	–	–	6 (8)	–	10 (5)	**<0.0001**	**<0.0001**
Partial response	71 (84)	22 (73)	7 (87)	–	–	26 (34)	–	126 (63)		
Pathological complete response	12 (14)	6 (20)	1 (13)	–	–	45 (58)	–	64 (32)		

*IHC* immunohistochemistry.

Significant *p* values are in bold.

The ASCO/CAP Group 2 patients (*n* = 46) showed pCR in 26% of tumours compared with 18% of those in Group 1 and 8% of those in Group 3, respectively (both currently categorised as HER2 positive in the UK) (Table 1). There was no significant difference in the response rate between Group 2 and Group 1 (*p* = 0.70) or between Group 2 and combined Groups 1 + 3 (*p* = 0.58). Similar results were identified when the cohorts were stratified according to HER2 targeted therapy or ER status; the response rate in Group 2 was not different to Group 1 tumours (*p* = 0.575 and *p* = 0.375 in the ER negative and ER positive cases, respectively) or combined Group 1 and Group 3 tumours (*p* = 0.73 and *p* = 0.44 in the ER negative and ER positive cases, respectively).

Group 2 tumours showed lower pCR rates compared to those in the HER2 IHC 3+ group (*p* = 0.001). This difference was maintained in the ER positive tumours (*p* < 0.0001) but not in ER negative cases (*p* = 0.42). Although the response rate of Group 2 tumours was higher than that of the combined Groups 4 and 5 (*p* = 0.021), this difference was not evident when the cohort was stratified based on ER status (*p* > 0.05). The pCR in patients with HER2 negative tumours (IHC 0 or 1+) receiving NACT was 21% (30% in ER negative and 13% in ER positive) compared with 27% of patients in Group 2 receiving NACT and HER2 targeted treatment (37% in ER negative and 20% in ER positive) (Table 2).

## Discussion

HER2 expression status is critical for selecting BC patients eligible for HER2 targeted therapies. Currently, IHC and FISH are regarded as equivalent assays for the assessment of HER2 status, due to their high concordance rate. Approximately 15–30% of BCs are IHC equivocal (2+), of which 15–30% are *HER2* amplified. IHC 2+ amplified tumours comprise ~20–40% of HER2 positive cases.^3,22,25,26^

HER2 protein, and not the gene amplification per se, drives BC growth and progression, and is blocked using targeted agents. Emerging evidence indicates that BC patients with strong protein expression (IHC 3+) benefit to a greater extent than those with gene amplification but with a borderline level of protein expression (IHC 2+ amplified).^27^ IHC 3+ cases almost always show high level gene amplification, whereas the IHC 2+ cases often show low level amplification and/or heterogeneity.^6,27–30^ Response to NACT plus anti-HER2 targeted therapy occurs more frequently in tumours with *HER2/CEP17* ratios >3.7 and *HER2* gene copy number >11.5.^28^ In a study of HER2-targeted therapy without chemotherapy, none of the 11 patients with *HER2/CEP17* ratio <4.0 and/or *HER2* gene copy number <10.0 achieved pCR, compared to 29% of patients with ratios >4 and/or *HER2* copy number >10.^31^ Although some studies failed to demonstrate an association between the degree of *HER2* gene amplification and response to adjuvant HER2 targeted therapies, the number of events in these studies was low.^20,29,32^

It is not surprising that BCs with *bona fide* HER2 positivity, as evidenced by protein overexpression (IHC 3+) and/or high-level gene amplification, are more dependent on HER2 to maintain their malignant phenotype and are more responsive to HER2 targeted therapy. This is also reflected in studies looking at gene expression analysis, with cancers belonging to the HER2-enriched intrinsic subtype showing higher pCR rates and the patients showing an improved event-free survival than those with non-HER2-enriched tumours.^33^ Supporting this, and in line with our results, Krystel-Whittemore et al. reported that BC with IHC 3+ HER2 protein over-expression showed significantly higher pCR rate (67%) compared to BC with IHC 2+ and *HER2* gene amplification (17%).^27^

A variable level of response of HER2 positive BC to NACT (with the same therapeutic agents) has been reported, with pCR rates ranging from 23 to 70% in various studies.^23,34,35^ Although the NACT pCR rate of the IHC 2+ BCs with evidence of *HER2* amplification does not appear to differ from that of HER2 negative cases (IHC 0/1+), which varies between 10 and 35%,^23,34,36^ the magnitude of benefit from the addition of anti-HER2 targeted therapy is more important. Our results indicate that both groups (IHC 3+ and IHC 2+ FISH amplified) benefit from the addition of anti-HER2 targeted therapy despite the difference in the overall pCR rates achieved in each group. In clinical practice, categorisation of BC with equivocal protein expression (IHC 2+) but with *HER2* gene amplification remains a challenge. Determination of the level, or pattern, of amplification associated with survival benefits from anti-HER2 therapies remains to be defined. There is an agreement that BC with *HER2* gene copy number ≥6.0 are categorised as HER2 positive and historically a ratio ≥2.0 alone was used to define HER2 positivity regardless of the gene copy number.^10,17,19^ However, there are tumours with *HER2/CEP17* ratio ≥2.0 and *HER2* copy number <6.0 and categorisation of these cases is difficult due to their rarity and consequent lack of evidence on treatment efficacy. The latest ASCO/CAP guidelines update published in 2018 re-classified these tumours into 2 groups: Group 1 (*HER2/CEP17* ratio ≥2.0 and *HER2* copy number >4.0) remained as HER2 positive but Group 2 (ratio ≥ 2.0 and *HER2* gene copy number <4.0) was changed to HER2 negative.

Applying the 2018 guidelines has resulted in an increase in the proportion of HER2 negative cases compared with the 2013 classification.^37–39^ Evidence of pathological response in the neoadjuvant setting as a surrogate end point for survival outcome is a reasonable option, as we hypothesised in this study. Our results show that both groups have an inferior pCR compared to IHC 3+ but have a similar pCR rate to each other, which is slightly higher than the pCR rate in HER2 negative cases receiving chemotherapy alone. In a recent study by Wang et al.,^40^ there was no significant difference between Group 2 (*n* = 30) and Group 1 (*n* = 100) tumours regarding the outcome in terms of disease-free or overall survival in patients treated with or without targeted therapy. These results are consistent with our study, which did not find a difference in response rate to NACT and HER2 targeted treatment between Group 2 and Group 1 or between Group 2 and combined Groups 1 and 3. In the study of Perez et al. (*n* = 794),^29^ 1.5% of cases with a ratio ≥2.0 were IHC 0/1+ and 10% were IHC 2+ whereas 88.5% were classified as 3+ for HER2 IHC regardless of the *HER2* gene copy number; this supports the earlier concept that cases with ratios ≥2.0 are typically HER2 positive. In the adjuvant setting, Press et al.^20^ did not find a benefit from adjuvant trastuzumab therapy in patients with Group 2 tumours. However, 92% of Group 2 patients in the study were classified as IHC 0 or 1+, which may explain the lack of benefit. In that study, the distribution of HER2 IHC score (0/1+, 2+ or 3+) in tumours with *HER2* copy number <4.0 and those with ≥4.0–6.0 was similar whereas the difference in the distribution was significantly different between ratios <2 and ≥2.0 with 47% of cases with a ratio ≥2.0 scored as 3+.^20^ Although 50% of cases with HER2 copy number ≥6.0 scored 3+ this was observed only when the ratio was ≥2.0 compared to 11% when the ratio was <2.0; further emphasising the impact of the *HER2/CEP17* ratios on HER2 protein expression.^20^

Our study confirms that in patients with HER2 positive BC who receive targeted therapy, ER status is important clinically. The best response is seen in IHC 3+ tumours regardless of ER status, likely reflecting the dominance of HER2 over the ER pathway. However, in patients with HER2 positive tumours that are IHC 2+ amplified, there is a significant impact of ER status on the response rate; 38% pCR rate in ER negative tumours compared to 15% in ER positive tumours, which may reflect the mixed effect of HER2 and ER pathway activation on BC growth and progression.^41–43^ When the analysis was limited to patients who had received chemotherapy and HER2 targeted therapy and the cohort was stratified based on ER expression, the response rate of Group 2 was not different to that of Group 1 tumours or of the combined Group 1 and Group 3 tumours. Interestingly, in the subgroup of patients with ER positive BC who received chemotherapy and HER2 targeted therapy, those tumours that were IHC 3+ showed the highest pCR rate (58%) compared to 14% in the IHC 2+/ISH amplified tumours (Groups 1 and 3) (or 15% if Group 2 was included; combined Groups 1, 2 and 3). In the ER negative subgroup of BC in women receiving chemotherapy and HER2 targeted therapy the difference was less marked (pCR rate of 53% for IHC 3+ versus 38% for IHC 2+/gene amplified, respectively). This again highlights the importance of making therapy decisions using both HER2 and ER status.

This study has some limitations. The number of cases in Group 2 was limited. The number of cases in Group 3 was also low (*n* = 9) limiting reliable statistical analysis for this group. This has been a limitation in other large studies, such as that of Press et al.^20^ in which only one BC was classified as Group 3 with an IHC score of 2+. The number of cases with IHC 2+ and high-level gene amplification was also limited, as might be expected. This may explain the low pCR rate observed in Group 1 in this study. However, the overall response rate of IHC 2+ amplified BC in our study was in line with others^27^ using either the updated guidelines^3^ (Groups 1 + 3) or the 2013 ASCO/CAP recommendations.^44^ In order to report on sufficient cases with these uncommon patterns of HER2 expression, the cancers in this series were collected from several centres. Although there are some potential differences in disease stage and therapeutic regimens adopted between centres, this reflects real world data and is, we believe, therefore clinically relevant. Ideally, the data should be collected as part of a prospective randomised trial. More significantly, perhaps, no survival outcome measures could be included in this multi-institutional retrospective study as patients were treated in recent years without sufficient follow-up time; we hope that this information will be collected prospectively.

In conclusion, the rate of pCR to NACT and HER2 targeted treatment of BC that is HER2 IHC 3+ is higher than for tumours that are IHC 2+ and *HER2* amplified. The rates of pCR were similar in the ASCO/CAP Group 1 and Group 2 tumours showing score 2+ HER2 protein expression. Although our data does not clearly refute the ASCO/CAP recommendation to exclude Group 2 from HER2 targeted therapy, it provides evidence that Group 2 with IHC score 2+ should be evaluated further with respect to eligibility for HER2 targeted therapy. Further investigation, including prospective randomised clinical trials of this group is warranted.

## Supplementary information

Supplementary Tables

## Data Availability

The authors confirm the data that has been used is available on reasonable request.
