# Supplementary material for: Retrospective observational study of HER2 immunohistochemistry in borderline breast cancer patients undergoing neoadjuvant therapy, with an emphasis on Group 2 (HER2/CEP17 ratio ≥2.0, HER2 copy number <4.0 signals/cell) cases
Source: Br J Cancer. 2021 Mar 24;124(11):1836–42. doi: 10.1038/s41416-021-01351-8 (PMC8144199; doi:10.1038/s41416-021-01351-8)
Supplement: Supplementary file 1 — Supplementary Tables [file 41416_2021_1351_MOESM1_ESM.docx]

**Supplementary Table 1: Degree of pathological response in subgroups based on HER2 targeted therapy and oestrogen receptor status.**

| **Variables** | **HER2 Groups** | | | | **P value**  **Chi square** | **Adjusted p value**  **(Bonferroni correction)** |
| --- | --- | --- | --- | --- | --- | --- |
|  | **IHC 2+** | | **Positive Control Group IHC 3+**  **No. (%)** | **Negative Control Group IHC1+/0**  **No. (%)** |  |  |
|  | **Group A**  **No. (%)** | **Group B**  **No. (%)** |  |  |  |  |
| **Whole cohort**  No response  Partial response  Pathological complete response |  | | | | **<0.0001** | **<0.0001** |
|  | 7 (4) | 22 (12) | 10 (7) | 37 (16) |  |  |
|  | 138 (77) | 147 (77) | 57 (39) | 147 (63) |  |  |
|  | 35 (19) | 21 (11) | 79 (54) | 49 (21) |  |  |
| **HER2 Targeted therapy not offered**  No response  Partial response  Pathological complete response |  | | | | **0.001** | **0.008** |
|  | 2 (9) | 22 (12) | 1 (13) | 37 (16) |  |  |
|  | 19 (82) | 147 (77) | 5 (63) | 147 (63) |  |  |
|  | 2 (9) | 21 (11) | 2 (24) | 49 (21) |  |  |
| **HER2 Targeted therapy given**  No response  Partial response  Pathological complete response |  | | | | **<0.0001** | **<0.0001** |
|  | 5 (3) | 0 (0) | 9 (6) | 0 (0) |  |  |
|  | 119 (76) | 0 (0) | 52 (38) | 0 (0) |  |  |
|  | 33 (21) | 0 (0) | 77 (56) | 0 (0) |  |  |
| **Oestrogen receptor negative**  No response  Partial response  Pathological complete response |  | | | | **0.003** | **0.021** |
|  | 1 (3) | 5 (12) | 4 (6) | 21 (19) |  |  |
|  | 22 (58) | 27 (64) | 26 (41) | 58 (51) |  |  |
|  | 15 (39) | 10 (24) | 34 (53) | 34 (30) |  |  |
| **Oestrogen receptor positive**  No response  Partial response  Pathological complete response |  | | | | **<0.0001** | **<0.0001** |
|  | 6 (4) | 17 (12) | 6 (7) | 16 (14) |  |  |
|  | 116 (82) | 120 (81) | 30 (37) | 87 (74) |  |  |
|  | 20 (14) | 11 (7) | 45 (56) | 15 (12) |  |  |

Cases were group as follows: Group A = ASCO/CAP Groups 1, 2 and 3 (HER2 2+, amplified), Group B = ASCO/CAP Groups 4 and 5 (HER2 2+, non-amplified), All tumours were selected from patients who received neoadjuvant therapy.

IHC; immunohistochemistry

Significant p values are **in bold**

**Supplementary Table 2: Proportional odd logistic regression model showing the association between various HER2 groups and other cofounders and the response to neoadjuvant therapy.**

| **Parameter** | **p-value** | **Hazard Ratio** | **95% Confidence Interval** | |
| --- | --- | --- | --- | --- |
|  |  |  | **Lower** | **Upper** |
| Tumour grade | 0.002 | 0.4 | 0.1 | 0.7 |
| Oestrogen receptor status | 0.282 | 1.3 | 0.7 | 2.5 |
| HER2 IHC 2+ / FISH Group 1 | 0.020 | 0.3 | 0.1 | 0.8 |
| HER2 IHC 2+ / FISH Group 2 | 0.040 | 0.2 | 0.1 | 0.9 |
| HER2 IHC 2+ / FISH Group 3* | N/A | N/A | N/A | N/A |
| HER2 IHC 2+ / FISH Group 4 | 0.577 | 0.7 | 0.2 | 2.3 |
| HER2 IHC 2+ / FISH Group 5 | 0.272 | 1.6 | 0.7 | 4.1 |
| HER2 IHC 3+ (Positive control) | <0.0001 | 0.2 | 0.1 | 0.3 |
| HER2 IHC 0 and 1+ (Negative control)* | N/A | N/A | N/A | N/A |

*The test was not computed for these 2 groups as the number of cases was either small or nil.

Significant p values are in **bold**

IHC; immunohistochemistry, FISH; fluorescence in situ hybridisation.
